# Design and Manufacture of a Micro-Ejector and the Testing Stand for Investigation of Micro-Ejector Refrigeration Systems

**DOI:** 10.3390/mi15040429

**Published:** 2024-03-23

**Authors:** Kamil Śmierciew, Dariusz Butrymowicz, Jerzy Gagan, Paweł Jakończuk, Mateusz Pawłowski

**Affiliations:** Department of Thermal Engineering, Bialystok University of Technology, 15-351 Bialystok, Poland; k.smierciew@pb.edu.pl (K.Ś.);

**Keywords:** microejector, supersonic ejectors, electronic cooling, ejector refrigeration systems, energy efficiency, waste heat, machining

## Abstract

This paper describes the procedure of design and manufacture of a micro-ejector proposed for miniature ejection refrigeration systems. It describes the procedure of design, fabrication, and experimentation on supersonic micro-ejectors and makes the case for isobutane as a working fluid for such systems. It was demonstrated that it is possible to design and fabricate a micro-ejector with a cooling capacity of approximately 3 W. The discussed micro-ejector was driven by a heat source with temperature below 60 °C. The evaporation temperature was approximately 15 °C. For these operating parameters, the reported entrainment ratio was approximately 0.20. The difficulties in fabricating the micro-ejector due to its small dimensions are discussed in the paper. Additionally, the potential difficulties and solutions related to ensuring and maintaining stable operation of the testing stand are presented. The performance of the proposed system is demonstrated and discussed, including relations between mass entrainment ratio, compression ratio, cooling capacity, and temperature.

## 1. Introduction

Miniaturization is a phenomena observed worldwide in electronic technology. The dimensions of electronic devices are becoming smaller while their computational power increases. In effect, the electricity consumption of these devices increases together with the density of heat generated. Increasing electricity consumption leads to an increase of greenhouse gases (GHG) [1], and the heat generated is also a problem because it has a negative effect on the efficiency of electronic devices. In order to eliminate or at least reduce the abovementioned problems, more and more sophisticated refrigeration systems are needed [2]. Today, mini- or micro-channel heat pipes provide one of the best, most practical methods of effectively operating cooling devices [3]. Another method, which is not as popular as heat pipes, is micro-ejectors. The micro-ejector, a compact device embodying the nozzle principle, finds its primary applications in vacuum processing, gas pressurization, and liquid spraying. Functioning akin to other nozzles, this miniature device lowers vapor pressure by increasing its velocity, facilitating gas pressurization, and vacuum treatment. It can be applied in micro-cooling systems, making it ideal for cooling micro-electronic devices, micro-sensors, and various other applications [4]. The micro-ejector assumes a pivotal role in micro-cooling systems, acting as the refrigerant injector for micro-cooling chips and other devices, thus achieving refrigeration. Moreover, it serves the purpose of heat recovery by dissipating heat from the micro-refrigeration device into the surroundings. As a control device in micro-cooling systems, the micro-ejector adeptly regulates airflow and temperature [5]. Through precise adjustments to the operational parameters of the micro-cooling system, one can attain a more accurate and efficient cooling effect. Concurrently, the micro-ejector boasts the advantages of a compact structure and low energy consumption, making it particularly suitable for micro-refrigeration devices and applications. Filippeschi [6] tested two types of miniature two-phase thermosyphons. The difference between these thermosyphons was in the evaporation section. One of them was designed for operation in horizontal orientation only and the second one operated both vertically and horizontally. The condenser and accumulation sections were the same for both types. An ultra-thin heat pipe with thermal capacity up to 6 W for cooling CPUs with a surface temperature up to 58 °C was proposed by Chen et al. [7]. Research studies aiming to improve the performance of heat pipes have been undertaken many times over the past decade. Special attention has been paid to the configuration of the flat evaporation section and the development and design of the wick [8,9,10,11]. Rakshith et al. [12] summarized the application of heat pipes in cooling applications for electronic devices and also demonstrated their abilities to enable effective cooling under dynamic conditions. As an alternative to heat pipes, cooling demands can be met by thermoelectric devices [13,14,15]. Heat pipes can be integrated with an ejector; according to Riffat and Holt [16], such systems provide very efficient performance and maintain very small dimensions. The results of operation of a combined ejector–heat pipe system are shown in [17,18]. Two geometric parameters are usually numerically tested using CFD; i.e., the ratio of the motive nozzle throat to the mixing chamber diameter and the motive nozzle exit position (NXP). The influence of these two parameters on the ejector performance of a capillary pump loop equipped with a micro-ejector was presented by Dong et al. [4]. Han et al. mentioned in [19] that the spontaneous condensation of motive steam within the nozzle not only diminishes the efficiency of the nozzle itself but also adversely affects the priming of the pumped fluid. Through numerical simulations, it was discerned that the pressure at the micro-nozzle outlet, computed using a wet steam model, aligned closely with predictions based on the ideal gas assumption. However, it was observed that the velocity magnitude was underestimated compared to the ideal gas predictions. This emphasizes the significance of refining the model for a more accurate representation. Furthermore, comprehensive studies including CFD and experimentation have been conducted on micro-ejectors, exploring their capacity to supply fuel–air mixtures for micro-combustion applications [20,21]. In [22], the authors investigated a high-powered thermal bubble micro-jet. An induction heater was used to enhance liquid delivery speed. This cutting-edge micro-ejector exhibited versatility for applications such as 3D printing, micro-scale liquid delivery, and micro-drug injection. Shifting the focus from traditional applications like toner manipulation and inkjet printing, Ben Hsieh et al. [23] took a pioneering approach. They developed a supersonic micro-jet device tailored for transdermal drug delivery. They found this approach was a safe and cost-effective method for administering vaccines with optimal efficiency.

To summarize the presented state of the art, it can be concluded that fabrication of miniature gas ejectors and problems related to manufacturing, such as surface roughness, accuracy of component dimensions, and their measurements, are for some reasons omitted in the scientific papers.

In this paper, the design and manufacture of a dedicated micro-ejector for a refrigeration system, as well as assessment of micro-ejector geometry, are presented.

The micro-ejector presented and described in this paper is intended for cooling systems of electronic equipment. The temperature of the heat source should not exceed 70 °C. Therefore, the aim of the present research was to design a miniature refrigeration system for cooling electronic devices using a single-phase vapor micro-ejector that met the above formulated criteria. The general objective was to define the micro-ejector geometry for given sets of operation parameters and working fluid, as well as to perform measurements on miniature ejectors to evaluate their performance.

**Figure 1:**
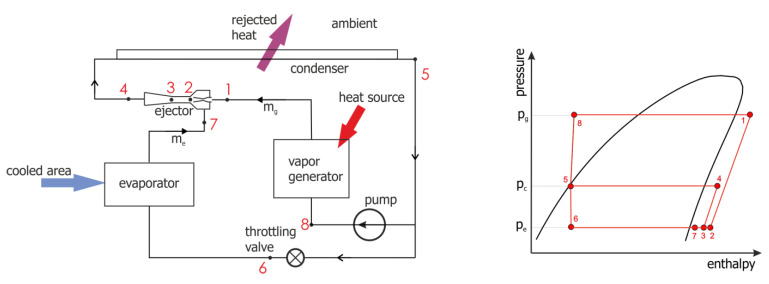
Schematic diagram of the ejector refrigeration system and its thermodynamic cycle in a pressure-enthalpy plot.

The schematic diagram of the discussed system is shown in Figure 1. Liquid refrigerant was passed through the pump to the generator (point 8). The liquid was heated in the generator by heat produced by the electronic equipment. The first stage of heating produced saturated vapor. With further heating, the vapor left the generator in a superheated condition and entered the ejector (point 1). The degree of vapor superheat was a function of the generator capacity and the mass flow rate. Such superheated vapor entered the ejector motive nozzle, where it underwent expansion from the generator pressure, *p_g_*, to a lower pressure, the evaporation pressure, *p_e_* (point 2). During this process, the ejector drew in vapor from the evaporator (point 7) and mixed it with the expanded vapor (point 2) to obtain a mixed vapor condition at point 3. Initially, the working fluid pressure rose slightly as a result of momentum exchange between the higher velocity gas entering the nozzle and the lower velocity gas being drawn in from the evaporator. Subsequently, the working fluid pressure rose further in the diffuser, reaching a maximum at the condensation pressure, *p_c_* (point 4). This compressed vapor entered the condenser, where it condensed. Depending on the cooling conditions in the condenser, the vapor may also subcool. The working fluid left the condenser in the liquid state (point 5). It was then divided into two parts: one part flowed to the generator through the circulating pump, while the remaining part flowed to the evaporator through the expansion (throttling) valve, which reduced (throttled) the pressure to the evaporation pressure, *p_e_*, achieving the condition of wet vapor (point 6). Through boiling in the evaporator, the working fluid absorbed cooling capacity, *Q_e_*, from the refrigerated medium and thereby cooled the substrate (the cooled portion of the electronic component).

The following operation parameters were assumed for a specific ejector cooling system:

- Evaporation saturation temperature: *t_esat_* = 12 °C;
- Generator temperature (saturation): *t_gsat_* = 65 °C;
- Ambient temperature: *t_amb_* = 25 °C;
- Evaporator cooling capacity: *Q_e_* = 3 W;
- Working fluid: R-600a, i.e., isobutane (which is a natural fluid and not a greenhouse gas).

## 2. Micro-Ejector Geometry Design and Manufacturing

The essential dimensions of the micro-ejector components were found using one of the most commonly applied approaches, proposed by Huang et al., using a 1D model [24]. With this model, the diameters of the nozzle and mixing chamber control the operation of the ejector with a double-chocked regime established. Isobutane was selected as a working fluid. Previous investigations [25] have proved that this refrigerant offers very promising and effective ejector operation and also provides environmental safety. In the next step, the CAD model was created and implemented in the CFD package software (Ansys 12). Prior to the CFD simulation, the hypothesis about micro-gas dynamics was evaluated. Experimental studies show that, in many cases, thermal-flow phenomena in microchannels cannot be explained using classical theories of fluid mechanics based on the continuum hypothesis. These include the laminar-turbulent transition, which begins much earlier in microchannels than in classical cases (i.e., =200) [26]. The relationship between flow resistance and the Reynolds number is also different than predicted by classical fluid mechanics. Those mentioned above and many other deviations from macro fluid mechanics are mainly due to the electrostatic effects of fluid–wall interaction, which cannot be neglected in the micro approach due to the large surface-to-volume ratio in such systems.

In general, there are two ways of modeling flows: the fluid is treated as a cluster of molecules or as the continuity is assumed, in which physical quantities are given and theoretically known at each point of the continuum. Continuum models fail when the scale of the phenomenon is comparable to the mean free path of the molecules. The ratio of these two quantities is called the Knudsen number and is defined as

$$Kn=\frac{\lambda }{L};$$

where *L* is the characteristic geometric dimension of the phenomenon (e.g., microchannel diameter), *λ* is the mean free path, which in turn for an ideal gas is given by

$$\lambda =\frac{kT}{\sqrt{2}\pi p\sigma^{2}};$$

where *k*—Boltzmann constant, *σ*^2^—particle diameter, for air 1.38 × 10^−19^ m, *T*—temperature [K], and *p*—pressure [Pa].

It is generally accepted that the classical continuum model, although with modified boundary conditions, is valid up to *Kn* < 0.1. The Navier–Stokes equation is valid when *λ* is much smaller than *L*. When this is not the case, the fluid is not in equilibrium and the linear relationship between stress tensor and velocity no longer holds. Similarly, the linear relationship between the heat flow and the temperature gradient is not valid. The commonly accepted methods of modeling as a function of the Knudsen number are as follows:

- *Kn* < 0.001; the flow is continuous (continuum flow C), classical fluid mechanics equation with classical (no-slip) boundary conditions;
- 0.001 < *Kn* < 0.1; the flow is continuous, but the boundary conditions must be modified by introducing a slip on the wall and a temperature jump (slip flow S);
- *Kn* > 10; the flow is considered as free molecular flow (H).

In the case presented in this paper, and assuming the lowest pressure and the average temperature of the isobutane as 0.15 MPa and 280 K, respectively, the average Knudsen number was equal to *Kn* = 3.47 × 10^−4^. Therefore, it was reasonable to consider the flow in terms of classical fluid mechanics.

The CFD technique was used to pre-test and evaluate the proposed geometry. The evaluation procedure required iterative actions; i.e., based on the obtained results the geometry was modified if needed and recalculated. By this means it was possible to avoid vortices or reducing the compression-expansion shock wave pattern at the nozzle exit, which leads to energy dissipation. However, CFD studies are not discussed in this paper. Once the proposed geometry was positively evaluated by CFD, the 3D CD model of ejector components was created. The tested mini-ejector was manufactured using conventional machining methods.

Because of the extremely small dimensions of the designed nozzle and conventional machining methods, it was necessary to fabricate several nozzles. However, only two of them had dimensions closest to the desired design dimensions. These nozzles were used in investigations. Nozzles are marked in this paper as (A) and (B).

In Table 1, the comparison between theoretical (design) and measured geometric parameters is presented.

In Figure 2, Figure 3, Figure 4, Figure 5, Figure 6, Figure 7, Figure 8, Figure 9, Figure 10 and Figure 11, the schematic diagrams and photos of fabricated elements are presented.

**Figure 12:**
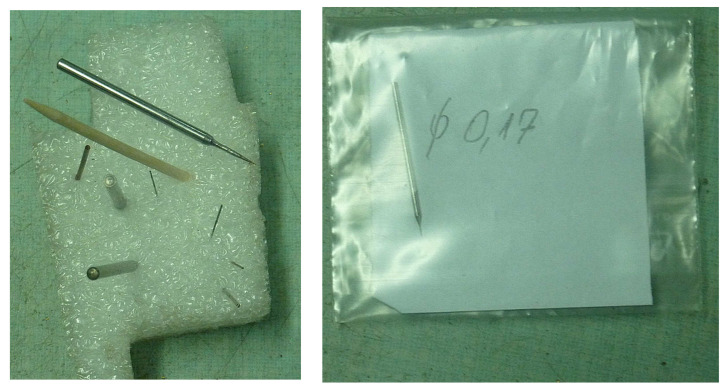
The **left** photo depicts a set of three holders for interchangeable drill tips of various diameters, also shown in the photo. The **right** photo illustrates the tool for diverging section fabrication (0.17 mm diameter).

**Figure 13:**
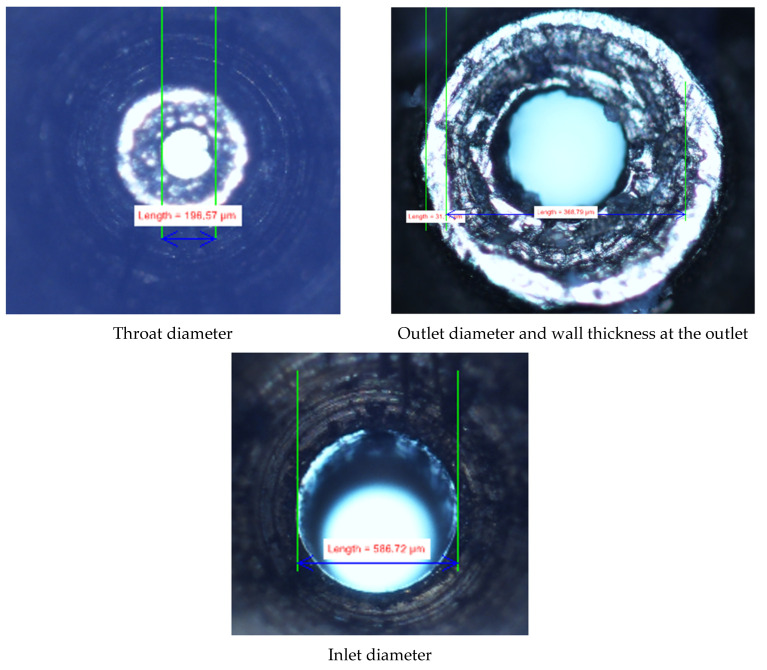
Photographs of nozzle A used for determining its geometry.

**Figure 14:**
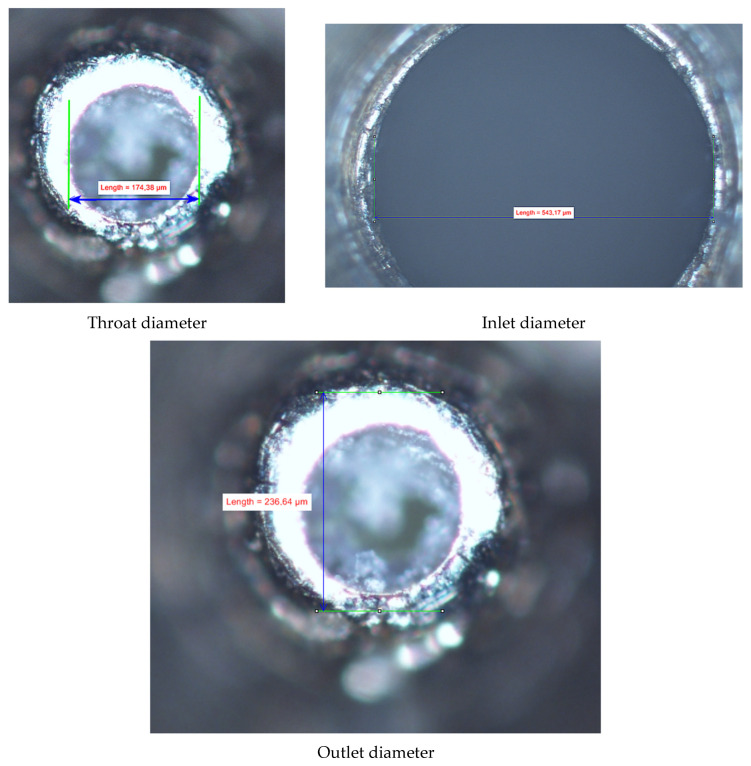
Photographs of nozzle B used for measuring its geometry.

Fabrication of the mini-ejector body was a challenge and required prefabrication of our own precision machining tools (shown in Figure 12), which achieved the appropriate accuracy of the geometry of individual parts of the ejector. The smallest diameter was in the throat of the motive nozzle. Another crucial part was the divergent part of the nozzle, its angle, surface roughness, and outlet diameter. As mentioned earlier, in order to obtain the best possible undisturbed flow pattern, the CFD technique was used for nozzle evaluation prior to manufacturing. Again, in order to obtain the required dimensions of the nozzle with relatively good accuracy, several nozzles were fabricated. Post-fabrication measurements determined those nozzles suitable for use in the tested mini-ejector. An optical microscope, the NIKON Eclipse TS-100F, was used for measurement of the nozzle dimensions. Additional 3D visualization, i.e., the measurements at different “depths” by changing the microscope focus, allowed for detailed assessment of the nozzle quality. Photos of the manufactured nozzle A are presented in Figure 13 and of nozzle B in Figure 14.

## 3. Testing Stand

**Figure 15:**
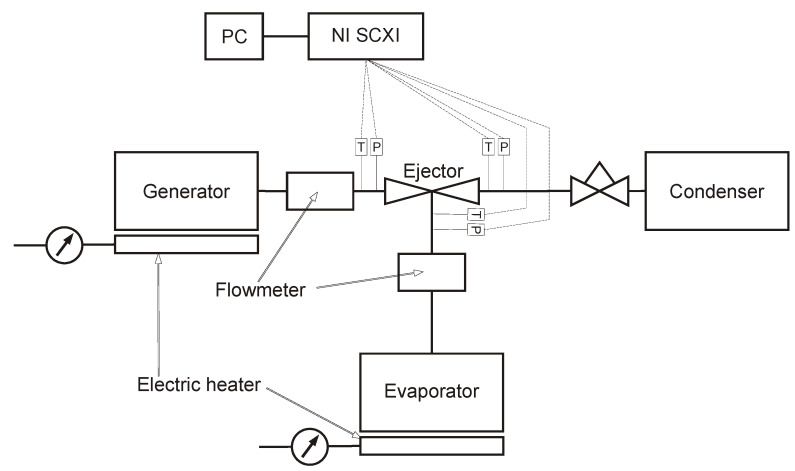
Schematic diagram of testing stand.

**Figure 16:**
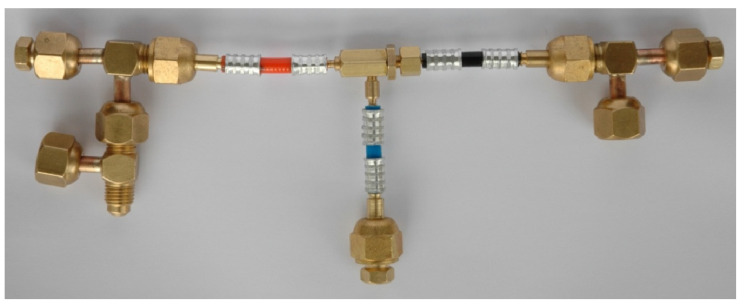
Photo of micro-ejector assembled with inlet and outlet connection ports.

The testing stand was designed to investigate the overall performance of the micro-ejector. An ejector refrigeration device is usually tested using different values of operating pressures and temperatures. For classic application, the motive source is usually defined and its operating temperature or range of temperatures is known. The pressure corresponds to the temperature. Therefore, the motive parameters are in most of the cases fixed. The parameters of the entrained vapor depend on the evaporator conditions, and usually they are defined by the user or by the type of application. However, it can be concluded that these parameters are also fixed. The outlet parameters, however, depend on the condenser cooling conditions, which in most cases are ambient, and these parameters change in the widest range. For this reason, the investigation of the system presented in the paper was conducted with constant motive and suction parameters and variable outlet parameters. Refrigeration systems operating with natural refrigerants have much higher leakage requirements compared to those with air. Therefore, we carefully tested the system prior to the experiment using a high-class electronic leak detector. During the experiments, pressure transducers with an accuracy of 0.25% of the full range were utilized. The motive stream parameters were measured using a pressure transducer with a scale of 0–2.5 MPa, while those at other locations used a scale of 0–1 MPa. Additionally, RTD temperature sensors (Pt100) were employed, with a measurement accuracy of 0.20% of the actual measured value. Furthermore, Coriolis mass flow meters with an accuracy of 0.15% of the measured value were utilized. The performance was indicated by the mass entrainment ratio and the compression ratio. Therefore, the mass flow rates of the refrigerant and the pressure at the inlets and outlet were required. Due to the extremely small dimensions of the micro-ejector and the technical problems related with connection of measurement devices and sensors with the micro-ejector itself, the pressure and temperature along the ejector were not measured. The schematic diagram of the testing stand is presented in Figure 15. A photo of the micro-ejector assembled with connection ports is shown in Figure 16.

The original design (Figure 16) of the measuring ports of the micro-ejector system consisted of ready-made commercialy available elements. Unfortunately, tee-type elements turned out to be an unfavorable solution due to the material they are made of. Stainless steel used in the tees led to destabilization of the measurements due to an unfavorable heat transfer coefficient. Before the measurements, the system was gradually heated up, but the stainless steel elements did not react like the rest of the system, which resulted in unstable conditions for measurement. The superheated vapor from the superheater supplied to the motive nozzle condensed in front of the micro-ejector, which actually stopped the flow and made it impossible to read the operating parameters correctly.

**Figure 17:**
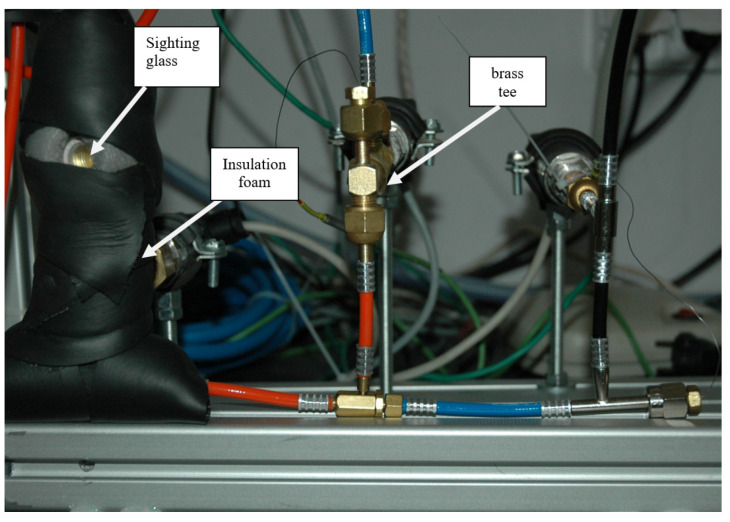
Changes in connection ports.

To eliminate this problem, elements made of brass were used, which was also the base material for the microejector. The motive nozzle and suction chamber connection ports were changed. The sensors and transducers were kept unchanged. Insulation foam was used to reduce heat losses to the environment (see Figure 17). Power cables were made of plastic and did not require additional insulation. Additionally, a sight glass was installed in the motive nozzle connection line, which enabled the identification of the refrigerant phase.

The entire system was equipped with an evaporator. A thermocouple connected to the evaporator red the wall temperature; measurements are included in the measurement table. Cold tap water was used to supply the evaporator, the temperature of which ranged between 8 and 14 °C. The condenser was cooled down by compressed air whose temperature was close to the ambient temperature.

An additional problem was maintaining the purity of the refrigerant and the corrosion resistance of the system. Due to the very small throat diameter of the experimental nozzle (0.18 mm), it was concluded that a foreign body such as a corrosion lump or oil drop may have caused a decrease in the efficiency of the nozzle and the entire ejector system. Therefore, it was important to keep the system clean as debris may have resulted in a lack of repeatability of measurements.

**Figure 18:**
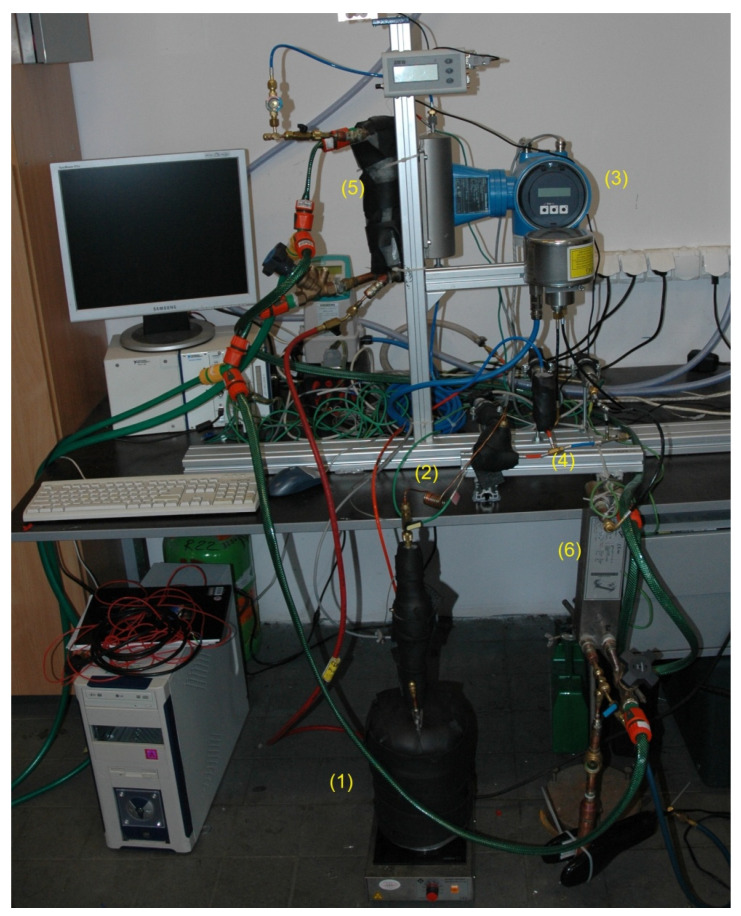
Final configuration of the test stand for determining the micro-ejector performance: 1—vapor generator; 2—vapor superheater; 3—mass flow meter; 4—test ejector; 5—evaporator; 6—condenser.

The final configuration of the testing stand is shown in Figure 18. The vapor generator consisted of a specially fabricated vessel (1) and an electric heater. The required motive nozzle operational parameters were achieved by controlling the heater electric power. Motive vapor at the required saturation pressure (and equivalent saturation temperature) flowed to the vapor superheater (2), which was a small-diameter tube heated by hot air from a blower. Superheating was controlled by regulating the hot air mass flow rate. This superheated vapor was delivered to the motive nozzle of the ejector under test (4). The motive vapor mass flow rate was not measured directly by a mass flow meter. The ejector sucked the secondary vapor from the evaporator (5). The secondary vapor mass flow rate was measured by a dedicated mass flow meter (3). The evaporation temperature was kept constant by means of controlling the temperature of cooled liquid (water) flowing to the evaporator. Compressed vapor flowed to the water-cooled condenser (6) and liquid isobutene then flowed to the collection vessel. Plate heat exchangers were used in both the evaporator and condenser.

## 4. Experimental Results

Two motive nozzles were used during experiments. The pressure in the vapor generator and in the evaporator was kept constant. The condensing pressure was varied by the change in air mass flow rate used for cooling the condenser. Averaged values of operating parameters for both nozzles are as follows:

Nozzle A: *p_g_* = 0.61 MPa, *p_e_* = 0.26 MPa, *t_gsat_* = 45.4 °C, *t_esat_* = 15.1 °C

Nozzle B: *p_g_* = 0.68 MPa, *p_e_* = 0.27 MPa, *t_gsat_* = 49.7 °C, *t_esa_*_t_ = 16.0 °C

where *p_g_* and *p_e_* are pressure in the vapor generator and evaporator, respectively, and *t_gsat_*, *t_esat_* are corresponding saturation temperature in the vapor generator and evaporator, respectively.

The performance parameters were defined as follows:

$$U—\mathrm{mass}\;\mathrm{entrainment}\;\mathrm{ratio},\;U=m_{e}/m_{g}$$

$$\Pi —\mathrm{compression}\;\mathrm{ratio},\;\Pi =\frac{p_{c}-p_{e}}{p_{g}-p_{e}}$$

$$Q_{e}—\mathrm{refrigeration}\;\mathrm{capacity},{\dot{Q}}_{e}={\dot{m}}_{e}\left(h_{e}-h\prime (t_{e})\right)$$

where *m_e_*—mass flow rate of the secondary vapor (kg/s); *h_e_*—specific enthalpy of the secondary vapor, *h_e_ = f*(*ρ_e_,t_e_*) (J/kg); *h*’(*t_e_*)—specific enthalpy of the saturated liquid at temperature, *t_e_* (J/kg), *p_c_*—backpressure (Pa).

**Figure 19:**
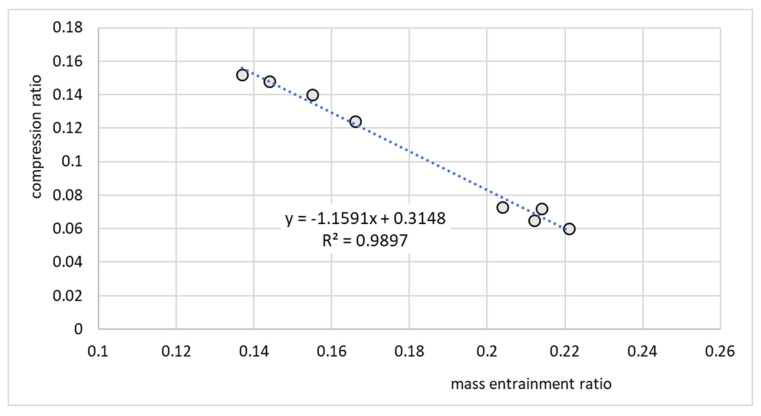
Performance line Π = *f*(*U*) of the ejector for the test nozzle A.

Figure 19 presents the ejector performance characteristics as a relationship between the compression and mass entrainment ratios for the micro-ejector equipped with nozzle A. Note that this relationship was well approximated by the linear function

Π=a⋅U+b

where, in this case, the coefficients were *a* = −1.1591 and *b* = 0.3148.

**Figure 20:**
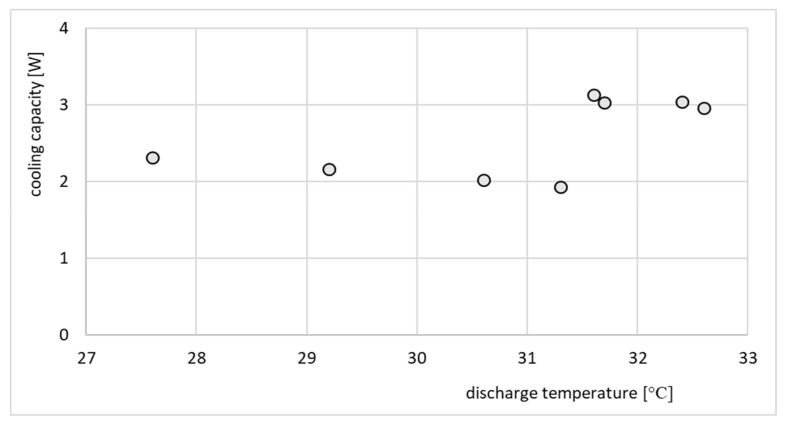
Refrigeration capacity, *Q_e_*, vs. discharge temperature for the test nozzle A.

The refrigeration effect of the same ejector is presented in Figure 20.

**Figure 21:**
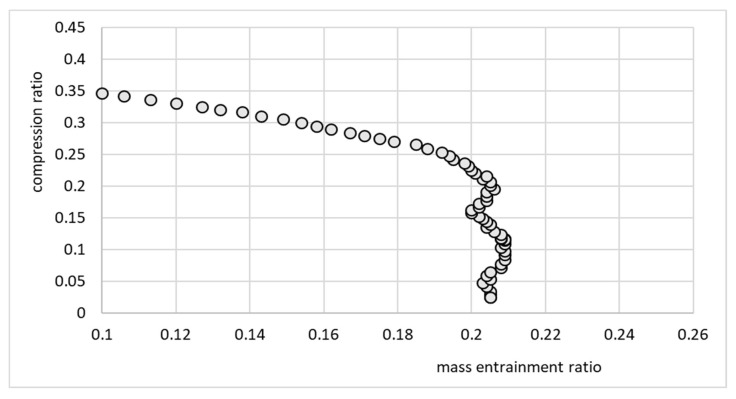
Performance line of the ejector fitted with motive nozzle B, test series B.2.

**Figure 22:**
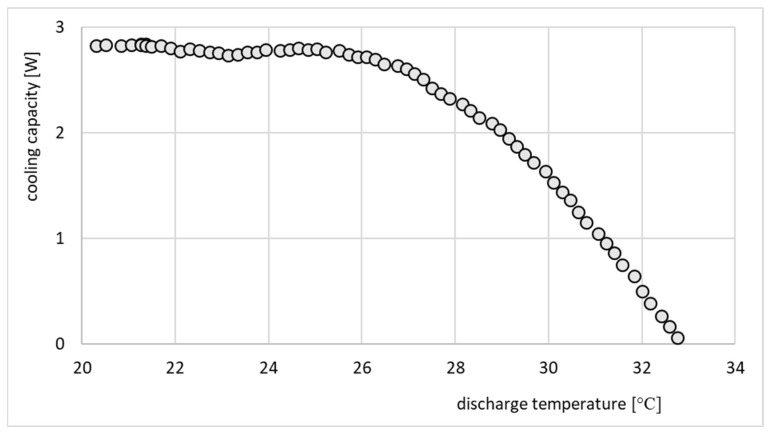
Refrigeration capacity vs. condensation temperature for the ejector fitted with motive nozzle B.

The performance line shown in Figure 19 indicates that the ejector operated in an off-design regime, since an increase in the compression ratio resulted in a decrease in the entrainment ratio. Such performance is typical for off-design operation. On the other hand, the performance of the ejector shown in Figure 20 cannot be considered as typical. The cooling effect was expected to gradually decrease as the discharge temperature and corresponding discharge pressure increased. In this particular case, the cooling capacity was roughly constant and between 2 and 3 W. This behavior, especially in terms of cooling capacity, was an effect of moderate variation of operating parameters, especially the motive pressure. Fluctuations of motive pressure influence the mass flow rate of the motive stream and further, the entrained stream. It is noteworthy that the investigation of the ejector equipped with nozzle A involved recording significantly fewer operating points, as depicted in Figure 19, Figure 20, Figure 21 and Figure 22. This limitation arose from the challenges in attaining stable operating conditions. Upon comparing the nozzle throat diameters, highlighted in Table 1, it became apparent that nozzle A was larger than nozzle B, deviating by 16% from the design value, whereas nozzle B only deviated by 3%. The substantial difference between the designed nozzle and the manufactured nozzle (A) resulted in a higher mass flow rate of refrigerant than expected. Consequently, the generator operated in an unstable manner, characterized by lower pressure and temperature. This instability prevented the ejector from achieving the double-choked operating mode. In contrast to the results of the nozzle A, the results of operation of the ejector equipped with nozzle B, shown in Figure 21 and Figure 22, indicate the steady state operation of the ejector. It is seen that constant refrigeration capacity was obtained and maintained as long as the ejector operated as double-choked. It was expected that for an on-design operating regime, the entrainment ratio would exceed the maximum value and remain constant with increasing in discharge pressure. Figure 21 shows that for an on-design operating regime, the mass entrainment ratio varied between 0.20 and 0.21. Such small changes may result from inevitable fluctuations in the parameters of the vapor generator and evaporator. Especially small devices are extremely sensitive to parameter fluctuations. In the case of test nozzle B, the refrigeration capacity was approximately 3 W with a critical condensation temperature of approximately +27 °C for on-design operation. It is suspected that the accuracy of the motive nozzle fabrication and the difficulty of determining the actual nozzle location were limiting factors in achieving the greatest possible refrigeration capacity (the optimum dimensions for the ejector body and nozzle could not be achieved precisely; those nozzles having dimensions closest to the optimum values were selected for evaluation). The better agreement between the throat diameter of manufactured nozzle B and the designed nozzle resulted in stable operation of the micro-ejector, providing a constant rate of heat exchange in the evaporator and vapor generator and better system control.

## 5. Conclusions and Recommendations

Based on the presented results, the following conclusions can be drawn:

1. It is possible to design and fabricate a micro-ejector using a 1D model;
2. For the discussed micro-ejector, the design refrigeration effect was 3 W and such values were achieved in experiments;
3. The maximum value of the entrainment ratio was slightly above 0.20 for both tested nozzles;
4. Difficulties in micro-ejector fabrication as well as problems related to maintaining the proper and stable operation of the entire system are discussed above.

## Figures and Tables

**Figure 2:**
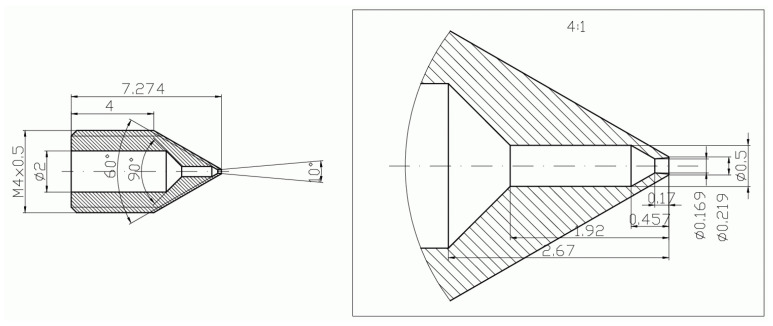
Schematic diagram of the motive nozzle.

**Figure 3:**
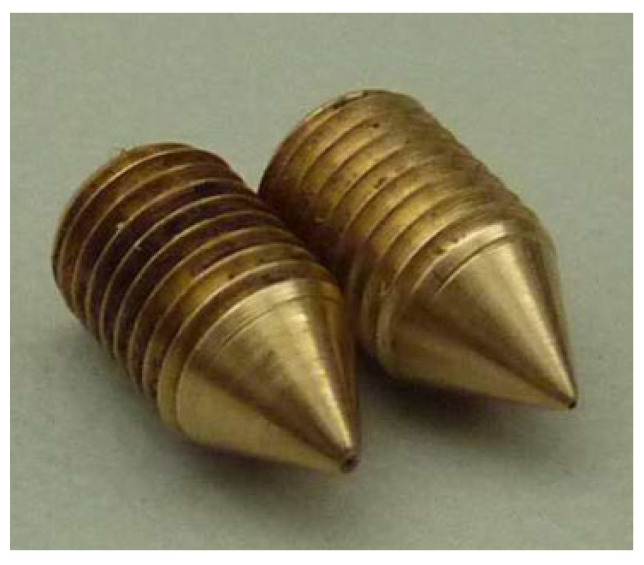
Photo of two fabricated nozzles.

**Figure 4:**
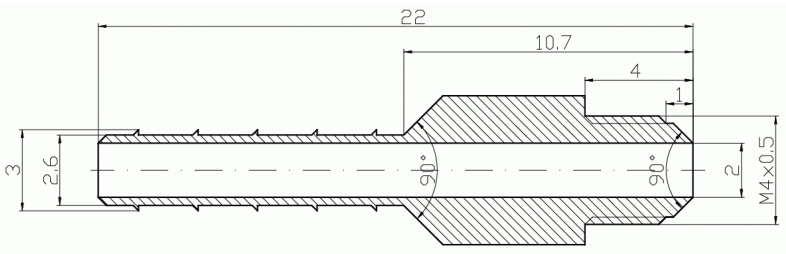
Schematic diagram of the motive nozzle connection port.

**Figure 5:**
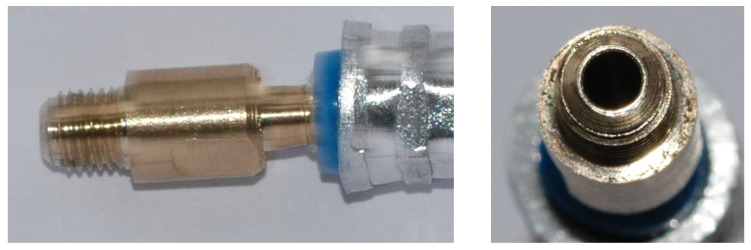
Photo of the nozzle connection port: side view (**left**); top view (**right**).

**Figure 6:**
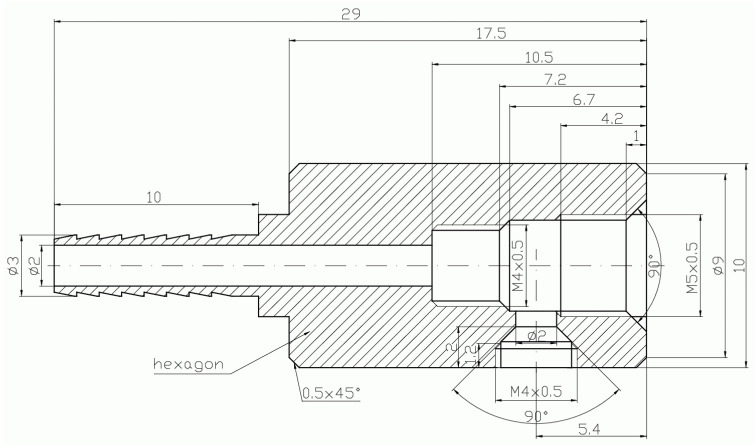
Schematic diagram of the suction chamber including connection port.

**Figure 7:**
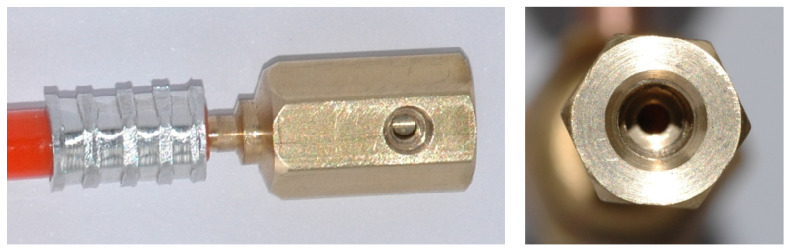
Photo of the suction chamber, including connection port: side view (**left**); top view (**right**).

**Figure 8:**
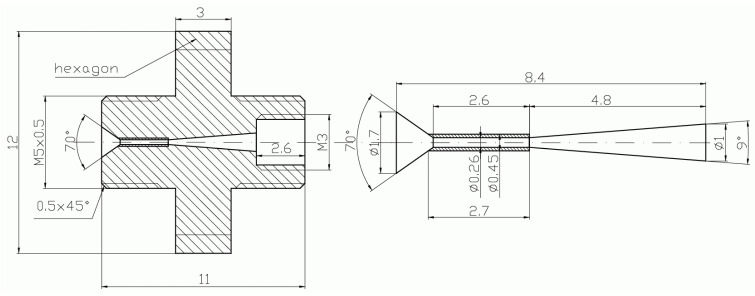
Schematic diagram of the mixing chamber with diffuser.

**Figure 9:**
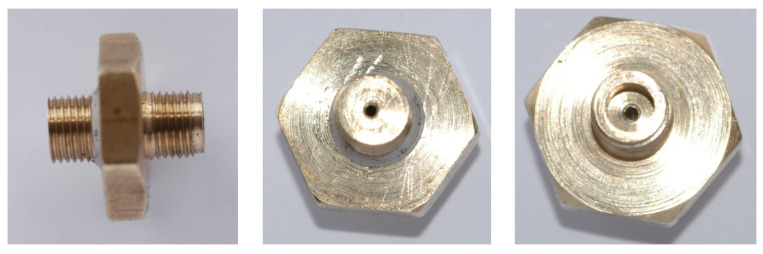
Photo of the mixing chamber with diffuser: side view (**left**); top view (**middle**, **right**).

**Figure 10:**
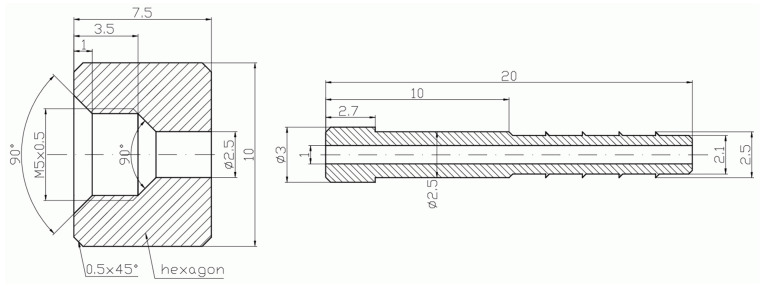
Schematic diagram of the diffuser connection port.

**Figure 11:**
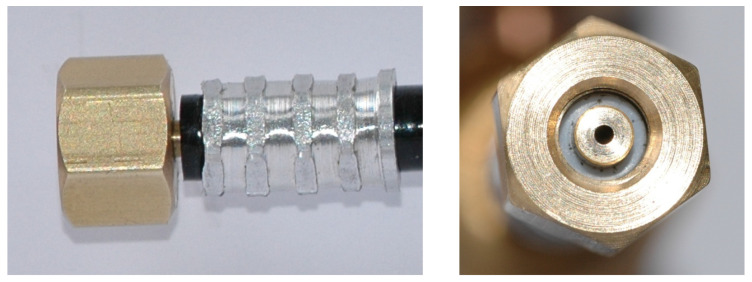
Photo of the diffuser connection port: side view (**left**); top view (**right**).

**Table 1:** Essential geometric parameters of the mini-ejectors to be fabricated.

Element	Design Parameter (mm)	Measured Parameter (mm)
Motive nozzle throat diameter	*d_cr_* = 0.169	0.196 (A); 0.174 (B)
Motive nozzle outlet diameter	*d_no_* = 0.219	0.368 (A); 0.236 (B)
Motive nozzle inlet diameter	*d_in_* = 0.5	0.586 (A); 0.543 (B)
Diameter of suction chamber inlet	*d_sin_* = 1.7	2.066
Mixing chamber diameter	*d_m_* = 0.26	0.251
Diffuser outlet diameter	*d_d_* = 1.0	1.047

## Data Availability

The original contributions presented in the study are included in the article, further inquiries can be directed to the corresponding author.

## References

[1] Belkhir L., Elmeligi A. (2018). Assessing ICT global emissions footprint: Trends to 2040 & recommendations. J. Clean. Prod..

[2] Zou L., Luo Y., Zhang J., Sheng X., Chen Y., Lin P. (2023). Phase change material gel particles with suitable size and superior thermophysical properties towards highly efficient thermal management of miniature electronic components. J. Energy Storage.

[3] Vasiliev L.L. (2008). Micro and miniature heat pipes—Electronic component coolers. Appl. Therm. Eng..

[4] Dong J.-M., Song H., Yu M.-Q., Wang W.-N., Pan X.-X. (2017). Numerical investigation of miniature ejector refrigeration system embedded with a capillary pump loop. Micromachines.

[5] Tashtoush B.M., Moh’d A A.-N., Khasawneh M.A. (2019). A comprehensive review of ejector design, performance, and applications. Appl. Energy.

[6] Filippeschi S. (2011). Comparison between miniature periodic two-phase thermosyphons and miniature LHP applied to electronic cooling equipment. Appl. Therm. Eng..

[7] Chen Z., Li Y., Zhou W., Deng L., Yan Y. (2019). Design, fabrication and thermal performance of a novel ultra-thin vapour chamber for cooling electronic devices. Energy Convers. Manag..

[8] Maydanik Y.F., Chernysheva M.A., Pastukhov V.G. (2014). Review: Loop heat pipes with flat evaporators. Appl. Therm. Eng..

[9] Gian P.C., Maurizio C., Massimo F. (2010). Experimental tests of a stainless steel loop heat pipe with flat evaporator. Exp. Therm. Fluid Sci..

[10] Yeh C.C., Chen C.N., Chen Y.M. (2009). Heat transfer analysis of a loop heat pipe with biporous wicks. Int. J. Heat Mass Transf..

[11] Choi J., Sung B., Kim C., Borca-Tasciuc D.A. (2013). Interface engineering to enhance thermal contact conductance of evaporators in miniature loop heat pipe systems. Appl. Therm. Eng..

[12] Rakshith B.L., Asirvatham L.G., Angeline A.A., Manova S., Bose J.R., Raj J.P.S., Mahian O., Wongwises S. (2022). Cooling of high heat flux miniaturized electronic devices using thermal ground plane: An overview. Renew. Sustain. Energy Rev..

[13] Liu H.R., Li B.J., Hua L.J., Wang R.Z. (2022). Designing thermoelectric self-cooling system for electronic devices: Experimental investigation and model validation. Energy.

[14] Sun X., Zhang L., Liao S. (2017). Performance of a thermoelectric cooling system integrated with a gravity-assisted heat pipe for cooling electronics. Appl. Therm. Eng..

[15] Di Capua H M., Jahn W. (2021). Performance assessment of thermoelectric self-cooling systems for electronic devices. Appl. Therm. Eng..

[16] Riffat S.B., Holt A. (1998). A novel heat/ejector cooler. Appl. Therm. Eng..

[17] Ziapour B.M., Abbasy A. (2010). First and second laws analysis of the heat pipe/ejector refrigeration cycle. Energy.

[18] Zhu L., Yu J. (2016). Simulation of steady-state operation of an ejector-assisted loop heat pipe with a flat evaporator for application in electronic cooling. Appl. Therm. Eng..

[19] Han Y., Wang X., Wang W., Lee Y.X., Li A. (2023). Numerical Investigation of Transonic Flow-Induced Spontaneous Condensation in Micro-Ejector Nozzles. Micromachines.

[20] Papadopoulos G., Tyll J., Drake A., Chue R., Williams J.D., Galambos P.C. Air Entrainment Studies for a Supersonic Micro-Ejector System. Proceedings of the Fluids Engineering Division Summer Meeting.

[21] Fan Y., Suzuki Y., Kasagi N. (2006). Development of a large-entrainment-ratio axisymmetric supersonic ejector for microbutane combustor. J. Micromech. Microeng..

[22] Hou Y., Liu B., Yang J. (2016). Research on a large power thermal bubble micro-ejector with induction heating. Microsyst. Technol..

[23] Hsieh H.B., Pattekar A., Uhland S., Völkel A., Recht M., Linn F., Anderson G., Chow E. Development of novel arrayed microjet devices for transdermal drug administration. Proceedings of the 40th Annual Meeting & Exposition of the Controlled Release Society.

[24] Huang B.J., Chang J.M., Wang C.P., Petrenko V.A. (1999). A 1-D analysis of ejector performance. Int. J. Refrig..

[25] Butrymowicz D., Śmierciew K., Karwacki J., Gagan J. (2014). Experimental investigations of low-temperature driven ejection refrigeration cycle operating with isobutane. Int. J. Refrig..

[26] Yener Y., Kakac S., Avelino M., Kakaç S., Vasiliev L., Bayazitoğlu Y., Yener Y. (2004). Single phase forced convection in microchannels. Microscale Heat Transfer Fundamentals and Applications.

